# Functional standing frame programme early after severe sub-acute stroke (SPIRES): a randomised controlled feasibility trial

**DOI:** 10.1186/s40814-022-01012-4

**Published:** 2022-03-03

**Authors:** Angela Logan, Jennifer Freeman, Bridie Kent, Jill Pooler, Siobhan Creanor, Doyo Enki, Jane Vickery, Andrew Barton, Jonathan Marsden

**Affiliations:** 1 Faculty of Health, School of Health Professions, University of Plymouth, Plymouth, UK; 2 Stroke Rehabilitation Unit, Royal Devon and Exeter NHS Foundation Trust, Exeter, UK; 3 Peninsula Clinical Trials Unit, Faculty of Health, University of Plymouth, Plymouth, UK; 4 Medical Statistics, Faculty of Health, University of Plymouth, Plymouth, UK; 5 Exeter Medical School, College of Medicine and Health, University of Exeter, Exeter, UK; 6 Faculty of Medicine & Health Sciences, University of Nottingham, Nottingham, UK; 7 NIHR Research Design Service, Peninsula Schools of Medicine and Dentistry, Plymouth, UK

**Keywords:** Stroke rehabilitation, Sub-acute stroke, Severe stroke, Supported standing, Task-specific training, Physiotherapy, Feasibility randomised controlled trial

## Abstract

**Background:**

Early mobilisation (> 24 h post-stroke) is recommended for people with stroke. However, there is a paucity of evidence about how to implement early mobilisation for people who have had a severe stroke. Prolonged standing and task-specific training (sit-to-stand repetitions) have separately been evaluated in the literature; however, these functionally linked tasks have not been evaluated in combination for people with severe sub-acute stroke.

**Methods:**

The objective was to determine the feasibility of conducting a randomised controlled trial (RCT) of a functional standing frame programme compared with usual physiotherapy for people with severe sub-acute stroke. An assessor-blinded feasibility RCT with nested qualitative component (interviews and focus group) and process evaluation was adopted. Participants were aged ≥ 18 years with new diagnosis of severe sub-acute stroke (modified Rankin Scale (mRS) 4/5) from four Stroke Rehabilitation Units across South West England. Participants were randomised to receive either: (1) functional standing frame programme (30 min. standing plus sit-to-stand repetitions) plus 15 min of usual physiotherapy daily (intervention); (2) usual physiotherapy (45 min) daily (control). Both programmes were protocolised to be undertaken a minimum of five sessions per week for 3 weeks.

Feasibility indicators included process, resource, management, and safety. Adherence, fidelity, and acceptability of the trial and intervention were evaluated using data recorded by therapists, observation of intervention and control sessions, interviews and one focus group. Patient measures of motor impairment, activities/participation, and quality of life were carried out by blinded assessors at baseline, 3, 15, 29, and 55 weeks post-randomisation.

**Results:**

Forty-five participants (51–96 years; 42% male, mRS 4 = 80% 5 = 20%) were randomised (*n* = 22 to intervention). Twenty-seven (60%) participants were followed-up at all time points. Twelve participants (27%) died during the trial; no deaths were related to the trial. Adherence to the minimum number of sessions was low: none of the participants completed all 21 sessions, and only 8 participants (18%) across both groups completed ≥ 15 sessions, over the 3 weeks; 39% intervention; 51% control sessions were completed; mean session duration 39 min (SD 19) control, 37 min intervention (SD 11). Intervention group: mean standing time 13 min (SD 9); mean sit-to-stand repetitions/session 5 (SD 4).

Interviews were conducted with 10 participants, four relatives and six physiotherapists. Five physiotherapists attended a focus group.

**Conclusions:**

The majority of progression criteria for this feasibility trial were met. However, adherence to the interventions was unacceptably low. This aspect of the trial design needs to be addressed prior to moving to a definitive RCT of this standing frame intervention in people with severe sub-acute stroke. Solutions have been identified to address these concerns.

**Trial registration:**

International Standard Randomised Controlled Trial Number ISRCTN15412695. Registration 19 December 2016.

**Supplementary Information:**

The online version contains supplementary material available at 10.1186/s40814-022-01012-4.

## Key messages

1. What uncertainties existed regarding the feasibility?

The feasibility of undertaking a RCT of a functional standing frame programme for people with severe sub-acute stroke as part of their inpatient rehabilitation was unknown, e.g. ability to recruit, randomise, train staff, deliver the intervention (and control) with required level of adherence/fidelity, maintain blinding, collect data.

2) What are the key feasibility findings?

The trial design was feasible in terms of recruitment, retention, ability to consent and consent rate, eligibility criteria, willingness of physiotherapists to recruit, acceptability of the intervention, burden, fidelity, orthostatic hypotension protocol and safety. However, adherence to the intervention and control was low, therefore, refinements to the intervention and control protocols are necessary before a definitive trial is planned.

The proposed outcome measures were deemed acceptable and most of the progression criteria for this feasibility trial were met.

3) What are the implications of the feasibility findings for the design of the main study?

Solutions for improving the design and delivery of a definitive trial have been identified to maximise the chances of its success in assessing the clinical and cost effectiveness of the functional standing frame programme. Adherence was low, therefore a systematic review will be conducted to determine how to optimise adherence in inpatient rehabilitation trials. This will contribute to the development of multi-modal training, which will incorporate clinical and personal equipoise, to optimise protocol adherence during trial set-up and throughout trial delivery. A pre-implementation checklist to assess levels of ‘on-board’ for sites and therapy departments, including staffing levels for future participating sites will be used.

## Background and objectives

Stroke is a sudden and devastating condition affecting over 100,000 people in the UK [1] and approximately 14 million people globally [2] per annum. The most common physical deficit caused by stroke is motor impairment [3] which can limit a person’s mobility across a wide range of daily activities: moving in bed; getting in/out of bed, on/off toilet, sitting out of bed, standing and walking [4]. These activities are particularly affected in the 15.5% of people with severe sub-acute stroke [1]. Thus, providing opportunities early after stroke to improve mobility by practising functional tasks, such as standing and moving between sitting and standing, are key focuses of rehabilitation [4–6].

Current concepts of biological recovery suggest a critical period of opportunity for neuroplasticity and repair [7] and that practising task-specific activities early after stroke can optimise recovery. People with a severe sub-acute stroke have limited options and opportunities to stand up and are reliant on physical assistance and equipment. Supported standing devices such as a motorised standing frame allow these individuals to attain and maintain a standing position through stabilising hips, knees and ankles with supports and/or straps [8]. Evidence from use in people with spinal cord injury, multiple sclerosis, stroke and traumatic brain injury indicates there are multiple benefits of supported standing programmes [9–15]. However, there are no evidence-based guidelines for implementing these standing programmes in adults with stroke, and evidence on effectiveness is insufficient [16] and contradictory [17]. For example, programmes vary in duration (20–60 min), frequency (3–5 time/week) and in the severity of the targeted population. Our study aimed to address some of the methodological limitations in previous studies. Additionally, the passive nature of prolonged supported standing warrants acknowledgement. It is possible that the addition of task-specific training, such as repeated sit-to-stand during the standing intervention, might result in better functional outcomes.

Task-specific, or repetitive-task, training is based on the principle that improving performance of a particular task requires it to have a functional goal [18] and be practised numerous times [19]. Sit-to-stand is one of the most frequently performed functional tasks of daily living. It is an essential pre-requisite to walking and important for independence in activities of daily living [20].

The functional standing frame programme in our feasibility trial combined two physiotherapy interventions that have been separately evaluated (task-specific training in people with mild to moderate stroke and prolonged standing in moderate to severe stroke). To our knowledge, this is the first study to explore these functionally linked tasks in combination for people with severe sub-acute stroke.

### Feasibility trial objectives

The objectives were to determine:

- Process: eligibility criteria, ability to consent, consent rate, recruitment rate, willingness/ability of physiotherapists to recruit and participants to be randomised, retention rate, acceptability of the intervention (participants and physiotherapists), determining usual physiotherapy and sample size estimates.
- Resource: burden to participants, treating physiotherapists and research assessors (e.g. factors arising from the trial and trial processes).
- Management: participant adherence to the intervention/control and trial, fidelity, acceptability of outcome measures (participants and physiotherapists), orthostatic hypotension (OH) protocol, feasibility of potential primary outcome measures (Barthel Index and Edmans ADL Index for Stroke).
- Safety: Number and nature of serious adverse events (SAEs) and adverse events (AEs) in both groups.

### Nested qualitative component objectives

The objectives of the qualitative evaluation were to determine:

#### Process

How trial procedures (timing and mode of participant recruitment, information provision, methods of data collection) can be refined to maximise recruitment, retention and acceptability in a definitive RCT; participants’ experience of the intervention, being randomised and reasons for, and experience of, withdrawing from the trial.

#### Burden

Physiotherapists’ attitudes, thoughts and feelings of the trial documentation and trial procedures.

#### Management

Relatives’ influence of participants’ decision to consent to participate, remain in the trial or provide assent for their relative; physiotherapists’ attitudes, thoughts and feelings of implementing the intervention.

## Methods

A pragmatic, multi-centre, assessor-blinded parallel two-arm feasibility RCT to determine the feasibility of a 3-week functional standing frame programme (prolonged standing and sit-to-stand repetitions) versus usual physiotherapy for people with severe stroke during inpatient sub-acute rehabilitation. A nested qualitative component and process evaluation were conducted.

### Trial setting

The trial was conducted in three healthcare sites, comprising four Stroke Rehabilitation Units (SRUs) based in two counties in the South West Peninsula of England. A full list of trial sites is available via the trial website [21].

### Process indicators

#### Recruitment

Consecutive patients admitted for inpatient stroke rehabilitation between 01 January 2017 and 30 September 2017 were screened for eligibility within 48 h of admission or being medically well for rehabilitation.

#### Consent

Written informed consent was provided if the person was deemed to have mental capacity [22]. A consultee provided written informed consent if participants lacked capacity to enrol in the trial.

### Eligibility criteria

Patients were eligible if they had a confirmed clinical diagnosis of new (first/recurrent) severe stroke, aged ≥ 18 years and graded as modified Rankin (mRS) 4 or 5. Full details of inclusion and exclusion criteria are given in the published trial protocol [23].

### Randomisation

After baseline assessment, participants were allocated (1:1) by computer-generated assignment to intervention or control group by the Peninsula Clinical Trials Unit (PenCTU). A minimisation procedure was used to minimise imbalance between groups with regard to both baseline fatigue and OH, using a bespoke, web-based system designed by the PenCTU. The minimisation algorithm included a random element, with probability of 0.9 for least imbalance allocation and 0.1 for other allocation. Fatigue was determined using a visual analogue scale (VAS), specifically chosen to enable people with aphasia to use, and dichotomised as fatigue (VAS: 4–10) versus no/minimal fatigue (VAS 0-3). OH was defined as a decrease in systolic blood pressure (BP) of ≥ 20 mmHg, or a reduction in diastolic BP of ≥ 10 mmHg when moving from a supine position to an upright posture, or from sitting to standing. Full details are given in the published trial protocol [23].

### Sample size

The target sample size was 50 participants, based on recommendations for feasibility studies [24] and justification given in the published trial protocol [23].

### Management indicators

#### Intervention and usual physiotherapy groups

##### Intervention

Functional standing frame programme (30 min standing plus sit-to-stand repetitions) plus 15 min of usual physiotherapy.

The intervention was protocolised to be delivered once a day for a minimum of five and maximum of seven sessions per week for 3 weeks. For a detailed description, see the Work Instruction [Additional file 1]. In brief, physiotherapists were requested to check participants’ BP for the first three sessions or until BP was within the participants’ normal range on three consecutive sessions. If a participant had a ≥ 20 mmHg drop in systolic BP and/or ≥ 10 mmHg diastolic BP within 3 min of moving from supine or sitting into standing, physiotherapists were directed to the OH protocol (see Additional file 1).

Each session was protocolised to last for 45 min to align with national UK recommendations [4]. This comprised 30 min (or as long as tolerated) using the standing frame (if required) which included standing and repeated sit-to-stand (up to 8–12 repetitions). An additional 15 min (or as long as tolerated) enabled practise of other activities deemed pertinent for discharge, such as transfers. The initial frequency and duration of standing was anticipated to vary according to physical capability; aiming to progress standing time and sit-to-stand repetitions by 30% in each session. If participants improved such that support from the standing frame was not required, they could progress to unsupported standing or walking for the remainder of the 3-week intervention period as well as undertake sit-to-stand repetitions within each 30-min session.

##### Control: usual physiotherapy (45 min)

Participants allocated to the control group received usual physiotherapy (routine stroke rehabilitation physiotherapy delivered in each SRU) for 45 min once a day (or as long as tolerated). Physiotherapists recorded activities undertaken during every session using the Physiotherapy Content Recording Tool developed specifically for the trial.

##### Fidelity

Fidelity was evaluated using two trial-specific standardised checklists outlining all components of the intervention and usual physiotherapy as per the study-specific Work Instruction (Additional file 1) plus space to record any protocol deviations. Five sessions covering both usual physiotherapy and intervention sessions were observed by an independent physiotherapist. Additionally, procedural fidelity was evaluated as part of the nested qualitative component.

##### Participant adherence

Adherence criteria were set for the intervention group only. Participants were deemed to have adhered to the functional standing frame programme if they completed all three components below:

1. A minimum of 15 or up to 21 sessions over the three-week intervention
2. Stood for 30 min per session (or 30% graded increase per session)
3. Performed 8–12 sit-to-stand repetitions (or 30% graded increase per session)

##### Clinical outcome measures

The following outcome measures were collected at baseline, 3, 15, 29 and 55 weeks post-randomisation:

- *Activities/participation*
• Barthel Index of Activities of Daily Living (BI) [25]
• Edmans Activities of Daily Living Index for Stroke Patients (Edmans) [26])
- *Motor impairments*
• Knee extensor muscle strength using a hand held dynamometer [27]
• Length of hip flexors, hamstrings and ankle plantarflexors using manual universal goniometer [28]
• Muscle tone in hip adductors, hamstrings and ankle using the Modified Ashworth Scale [29])
• Control of trunk using the Trunk Control Test [30]
• Fatigue using a visual analogue scale
- *Quality of life*
• Mood, assessed using the Patient Health Questionnaire (PHQ-9) [31] or Stroke Aphasia Depression Questionnaire-10 (SADQ-10) [32]
• Health related quality of life, assessed using the Stroke and Aphasia Quality of Life Scale-39 [33] and the EQ-5D 5L [34]

There were two assessors, each collecting all outcome measurements for their allocated participants. Face-to-face training was provided to optimise reliability of motor impairment assessment; however, inter-rater reliability between assessors was not formally captured. Standardised scripts were used for capturing the patient-reported outcome measures. Two proposed primary outcome measures (BI and Edmans) were used to determine if one appeared more sensitive to change in people with severe stroke.

Participant characteristics (age, gender, pre-stroke mobility, number of days since stroke, presence/severity of fatigue, presence of orthostatic hypotension) were collected at baseline. The target was to complete the baseline assessments within seven days of consent and all other assessments within ± 7 days of the pre-determined assessment dates, calculated from the date of randomisation.

##### Safety indicators

AEs and SAEs were documented by treating physiotherapists in the Case Report Forms for both groups during the intervention period and by the blinded assessor during the follow-up period. AEs and SAEs were collected via observation and clinical examination during the 3-week treatment period and recorded by treating therapists. Participants were prompted during follow-up visits and assessors recorded and reported AEs and/or SAEs. Occasionally treating therapists and/or assessors were alerted to AEs and/or SAEs after the 3-week period and discharge from SRU by non-trial staff who were part of the participants' treating team in a community or inpatient setting. AEs and SAEs were classified using the Medical Dictionary for Regulatory Activities (MedDRA) system [35].

### Qualitative component

Semi-structured face-to-face interviews with participants (*n* = 10), their relatives (*n* = 4) and the physiotherapists delivering the trial (*n* = 6) were conducted by the lead author (AL). Purposive sampling was used to exclude severe aphasia and cognitive impairment and ensure representation of each SRU. Participants were offered the option of being interviewed individually or with their relative. Patient participants were offered the opportunity to be present during their relative’s interview. Physiotherapists were interviewed individually throughout the recruitment period to address several uncertainties or unknowns and inform the design and implementation of a definitive trial. A focus group with physiotherapists (*n* = 5) was conducted five weeks after recruitment closed to discuss their experiences to further evaluate and improve procedures for a definitive main trial. All interviews and focus group were held in a private and quiet environment. The topic guide for interviews and focus group was based on the qualitative component objectives (see introduction).

### Analytical methods

A detailed statistical analysis plan was published on the trial website [21] prior to database lock. Descriptive analysis of quantitative data was undertaken using SPSS (Version 24) [36], with mean between-group differences and corresponding 95% confidence intervals calculated for all outcome measures. Interview and focus group data were analysed using thematic analysis [37, 38]. NVivo [39] was used for inductive coding. Manual processes were used to search, review, define and develop themes. The Consolidated Criteria for Reporting Qualitative Research (COREQ) checklist was used to ensure complete and transparent reporting [40].

## Results

### Participants

**Fig. 1:**
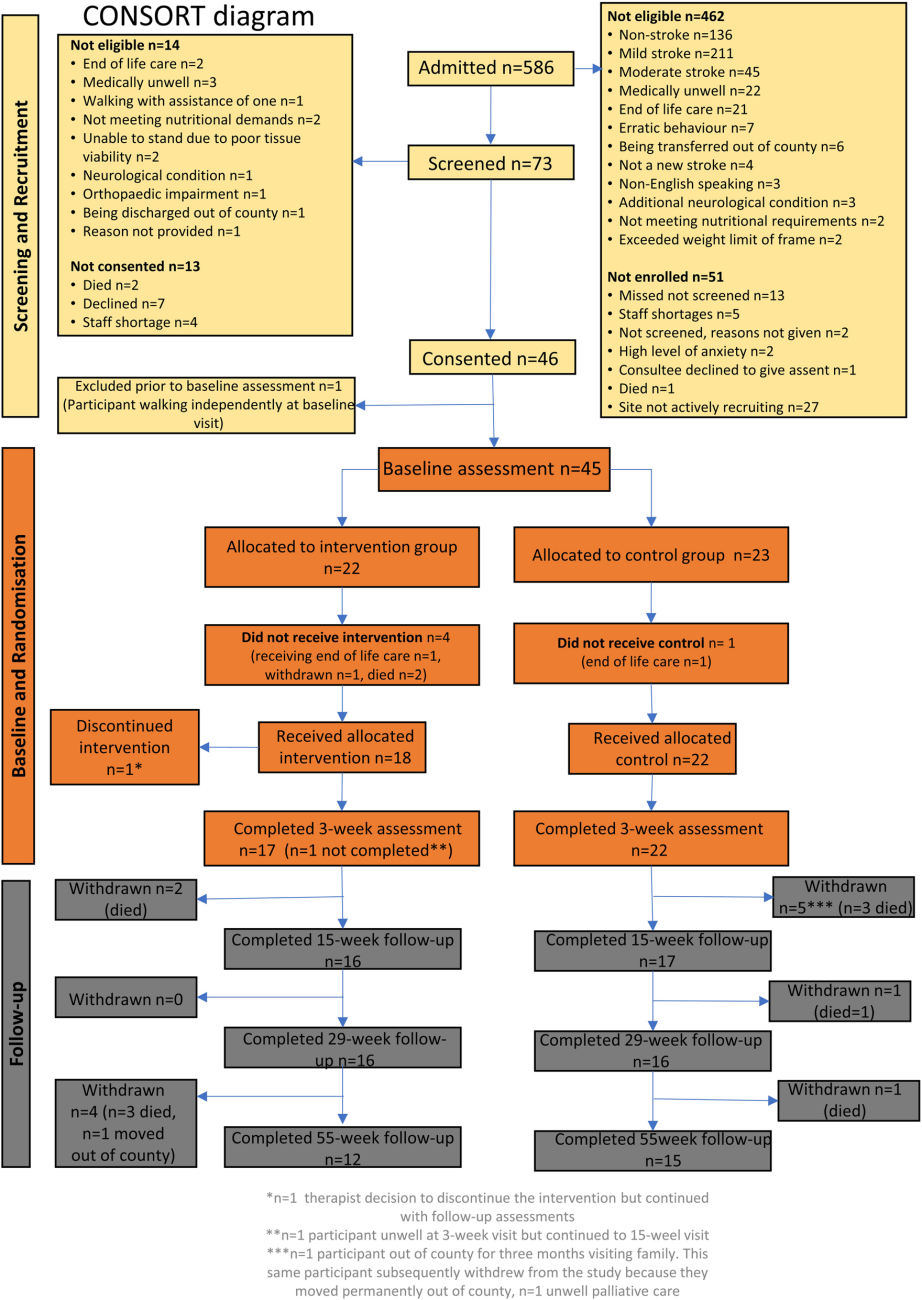
CONSORT flow diagram

The flow of participants through the trial, recruited between 1st January 2017 and 30th September 2017, is shown in the CONSORT [41] flow diagram (Fig. 1)

Forty-five participants were recruited (mean 4.5 participants per month), 90% of the recruitment target. Table 1 shows the baseline characteristics of the two groups, which were well balanced.

**Table 1:** Participant characteristics and baseline scores by treatment group

Characteristics	Intervention [*n* = 22]	Usual physiotherapy [*n* = 23]
**Age in years, mean (SD) [range]**	81.7 (11.7) [51–96]	78.9 (10.5) [60–94]
**Gender,** ***n*** **(%)**		
Female	12 (54.5)	14 (60.9)
**Days post-stroke participant was admitted to Stroke Rehabilitation Unit, median (range) [IQR]**	10.0 (0–31) [11]	6.0 (0–36) [10]
**Fatigue,** ***n*** **(%)**		
0–3 (“not tired”, “a little tired”)	4 (18.2)	4 (17.4)
4–10 (“tired”, “really tired”, “so tired I cannot do any more”)	18 (81.8)	19 (82.6)
**Orthostatic hypotension at randomisation**		
*n* (%) with orthostatic hypotension	4 (18.2)	3 (13.0)
**Modified Rankin Scale,** ***n*** **(%)**		
4 (moderately severe disability)	17 (77.3)	19 (82.6)
5 (severe disability)	5 (22.7)	4 (17.4)
**Barthel Index total score, mean (SD) [range]**	2.3 (2.06) [0–8]	2.6 (2.57) [0–10]
**Edmans Activities of Daily Living Index for stroke subgroup total, mean (SD) [range]**		
Washing	0.7 (0.50) [0–2]	0.6 (0.66) [0–2]
Grooming	2.2 (2.99) [0–9]	2.6 (3.10) [0–9]
Dressing	0.3 (0.72) [0–3]	0.4 (0.72) [0–3]
Meal times	2.9 (3.01) [0–9]	3.4 (3.28) [0–9]
Basic mobility	0.7 (0.76) [0–2]	0.7 (1.05) [0–4]
Advanced mobility	0.1 (0.21) [0–1]	0.1 (0.21) [0–1]
Bed mobility	0.1 (0.64) [0–3]	0.2 (0.74) [0–3]
Kitchen activities	0.1 (0.21) [0–1]	0.1 (0.21) [0–1]
Housework activities	0.0 (0) [0]	0.0 (0) [0]

### Consent

Twenty-nine participants (63%) provided written informed consent and 17 (37%) consultees declared written informed assent.

### Adherence

#### Number of sessions

Four-hundred and twenty-nine (45.4%) sessions were completed, 503 (53.2%) were not completed and information was missing on 13 (1.4%). The most common reasons for sessions not being completed were staffing (*n* = 264, 52%) and patients being unwell (*n* = 97, 19%). Twenty-two participants declined 53 sessions (10%) (32 sessions in the intervention group and 21 in the usual physiotherapy group (Table 2)).

**Table 2:** Summary of adherence to standing frame intervention for *n* = 22 participants

	Recommended per participant	Actual based on 180 sessions completed mean (SD)	Actual based on 180 sessions completed median [range]
**Number of sessions**	15-21	8.0, (5)	9.0 [1–16]
**Duration of session in minutes**	45	37 (11)	40 [5–60]^b^
**Standing time in minutes**	30 per session^a^	13 (9)	11 [1–35]
**Sit-to-stand repetitions**	8 to 12^a^	5 (4)	3 [0–20]

^a^or a 30% increase per session

^b^*n* = 7 (3.9%) of these were greater than 45 min

No participant completed all 21 sessions and only eight participants across the two groups completed 15 or more sessions over the three weeks: three (14%) in the intervention group and five (22%) in the usual physiotherapy group. Thus, during the trial, participants were not receiving the nationally recommended number of sessions for their stroke rehabilitation [4]. The mean number of sessions during the three-week physiotherapy period was 8.0 (SD 4.7) for the intervention group and 11 (SD 4.5) in the usual physiotherapy group, ranging from 1 to 16 for both groups.

#### Standing time and sit-to-stand repetitions

Table 2 presents summary statistics for duration of standing time and sit-to-stand repetitions for the intervention group. Not all participants in the intervention group stood in their sessions. Five participants across eight sessions did not stand at all during their documented intervention session.

### Fidelity

An independent assessor observed five sessions across three SRUs, completing a fidelity checklist (one SRU stopped recruiting after two months due to significant staffing issues); four sessions were delivered per protocol, one intervention group session deviated from the protocol (Table 3). Adherence was defined as the percentage of agreement between the checklist and what was observed during the session.

**Table 3:** Fidelity summary statistics

Fidelity criteria	***n***	% adherence
***Intervention group (n = 2 sessions observed)***		
Blood pressure checks	1	50
Use of standing frame or undertook prolonged standing outside the frame	2	100
Use of foot sensors in standing frame^a^	1	50
Use of straps and knee blocks in standing frame	1	50
Sit to stand repetitions	2	100
Documentation of standing time, adverse events, usual physiotherapy activities in the Case Report Form	2	100
Physiotherapists conducts the brief interview	1	50
***Control group (n = 3 sessions observed)***		
Physiotherapist undertook usual physiotherapy activities	3	100
Documentation of usual physiotherapy activities, adverse events, adherence to trial protocol (e.g. not using the standing frame) in the Case Report Form	3	100

^a^Foot sensors were supplied to all sites to enable physiotherapists to monitor the patient’s weight distribution during standing and sit to stand based on recommendations [17, 42]

### Physiotherapist and participant reflections

Ninety-one percent of sessions were enjoyed by participants. Full details of participants’ and physiotherapists reflections are not included due to word constraints.

#### Safety

Tables 1 and 2 (Additional file 2) provide a summary of AEs and SAEs reported during the trial. The highest number of AEs was reported at 55 weeks. Two SAEs (both deaths) occurred during the 3-week treatment period, both in the intervention group. The biggest number of SAEs in both groups was infections. Infections, recurrent stroke and prolonged/required hospitalisation were expected in people with severe sub-acute stroke. None of the AEs or SAEs were deemed related to the trial.

#### Proposed patient-centred outcome measures

Completeness of data for the proposed primary outcome measures (Barthel Index and Edmans) for the 27 participants who completed the trial was excellent (Table 3, Additional file 6); all but one participant completed these outcome measures. However, 12 participants died and six were withdrawn (Table 2, Additional file 2).

Mean differences between groups, and corresponding standard deviations, between baseline and 3, 15, 29 and 55 weeks for the proposed primary outcomes are presented in Table 2. Barthel Index scores improved at each time point in both groups, with the highest change score in the intervention group at 55 weeks. Bigger mean changes in the intervention group for some domains in the Edmans were observed between baseline and three weeks, which was in contrast with the usual physiotherapy group (Table 4, Additional file 4).

#### Ability to self-report proposed primary outcome measures

Cognitive and/or communication impairment affected some participants’ ability to self-report for the Barthel Index and Edmans, and in such instances a proxy was used (clinician, relative or carer). The proportions of patient participant and proxy responses for these measures are shown in Fig. 1, Additional file 7. Ability to self-report was the same for both measures, and similar across time-points: 19 of the 45 participants (42.2%) at baseline and 22 (48.9%) at 55 weeks.

##### Proposed secondary outcome measures

Completeness of data for the patient-reported outcome measures (PROMs) (Additional file 3) was affected by participants’ cognitive and/or communication impairment(s). For those unable to self-complete the PHQ-9, this was replaced by the SADQ-10 (observational measure) completed by a clinician, carer or relative.

#### Assessment intervals

Encouragingly 44 (97.8%) participants completed baseline assessments within our target of 7 days of consent and within ± 7 days of pre-determined assessment dates. This completion rate remained high for all visits, with 92.6% of those who remained in the trial attending their 55-week assessment within the pre-specified window.

#### Blinding

Outcomes were measured by two assessors due to constraints of the funding period. Assessor 1 (CI) was unblinded (as intended) to 10 participants after the 3-week assessment for interview purposes. The authors acknowledge the potential influence this may have had on subsequent follow-up assessments; however, changes between the 3-week and 3-month assessment were similar for assessors who were/were not unblinded. Assessor 2 maintained blinding as intended. The number of instances the blinded assessors guessed group allocation correctly was higher than chance at each of the follow-up visits: 66% 3 weeks; 76% 15 weeks; 69% 29 weeks; 74% 55 weeks.

#### Qualitative component

Ten participants (*n* = 4 intervention; *n* = 6 usual physiotherapy), four relatives and six physiotherapists were interviewed from across all four SRUs. Two of the six physiotherapists who were interviewed individually also attended the focus group at the end of recruitment along with three other physiotherapists not previously interviewed.

Described below are the four main themes derived from the data, which reflect the underlying objectives of the trial (see Table 8 for illustrative quotes).

1. ***Impact of organisation/culture on trial implementation***

Findings revealed that staffing resources were limited in some SRUs, the pressure for early hospital discharge was ever present and change or restructuring of services a constant feature. Nestled within these organisations were experienced and inexperienced physiotherapists struggling to deliver a protocolised feasibility trial in departments with a limited research culture and infrastructure, whilst maintaining equity of care for all patients. Physiotherapists identified that trial procedures competed with organisational priorities, such as pressure to discharge and the routine clinical admission assessments.

2) ***Impact of stroke on participation in the trial***

Patients and relatives spoke candidly about the devastating impact of stroke, for some there was a sense of impending death. Both parties stated they were trying to understand and come to terms with life after stroke, dealing with the physical and emotional consequences as well as social, occupational and financial issues that can arise. All these issues have the potential to affect recruitment and retention in clinical trials. Despite this, participants were willing to participate fully in the trial procedures. Hopes of recovery and return to their previous functional abilities, and altruism, were predominant factors influencing recruitment and retention.

Physiotherapists’ perception of the impact of stroke and using the standing frame with patients were often at odds with the patient perspective. Whilst physiotherapists reported patients were “too tired” [Physio 1] or “could not tolerate it” [Physio 6] patients described themselves as able to cope and “push through the tiredness” [Patient 4]. Both patients and their relatives emphasised that despite this devastating and life-changing event, patients should still be offered the opportunity to be involved in the trial and encouraged to practise standing. The discussions revealed, however, that physiotherapists acted as gatekeepers, deciding if and how the functional standing frame intervention was incorporated into the rehabilitation programme, based on their own beliefs/preferences.

3) ***Experience of trial procedures***

There were mixed opinions regarding trial procedures among physiotherapists; eligibility criteria and the “ideal patient” [Physio 5] for the intervention being the most widely debated topics. Physiotherapists were accepting of the trial design, however, there was no agreement about the trial duration. Most preferred shorter/fewer sessions per week, although only a few acknowledged the impact this would have on intensity of practice. Physiotherapists emphasised that the standing intervention was not suitable for every patient and recognised how their beliefs about the ideal patient affected recruitment and delivery of the intervention.

4) ***Patients’, relatives’ and physiotherapists’ experience of the functional standing frame intervention***

Some physiotherapists appeared to adopt a paternalistic approach, commenting patients were unable to tolerate the standing intervention or found it boring; this negatively affected recruitment and intervention adherence. For instance, the process data demonstrated, and physiotherapists commented that there were times when they violated the protocol by not screening or approaching potentially eligible participants. It was also evident from the focus group discussion that physiotherapists may not have always progressed or encouraged patient participants as much as they would if they were implementing an intervention that aligned with their beliefs, values or pre-conceived ideas about the ideal patient for this intervention. These factors could impact negatively on the results of a subsequent effectiveness trial. In direct contrast, some patients commented that they wished to be encouraged and supported to participate in challenging physical activities, despite feeling exhausted (Table 5, Additional file 5).

#### Assessment of pre-specified progression criteria

Evidence to support potential progression to a full trial was assessed against the three pre-specified criteria (Table 6, Additional file 6).

The pre-specified threshold for the recruitment target was met (green), whilst the rate of completion of follow-ups fell within the amber/scenario 2. The proportion of intervention participants categorised as adhering to the protocol (number and duration of sessions) did not meet the criteria for progression.

## Discussion

The primary aim was to evaluate the feasibility of undertaking a future definitive RCT, comparing a functional standing frame programme with usual physiotherapy for people with sub-acute severe stroke. The trial is not feasible in its current design. However, potential solutions have been identified to address a range of identified challenges that could be implemented into the design and delivery of an effectiveness trial for this under-represented patient group.

It was feasible to recruit participants with severe stroke during sub-acute rehabilitation (recruitment rate 4.5 participants per month). Unfortunately, recruitment started later than planned due to an unexpected delay starting the trial due to the HRA approval process being implemented in 2016. However, recruitment at one site was stopped < 2 months after opening due to significant staffing issues. A flexible recruitment rate at each site to reflect the number beds and therapists employed is recommended. It was apparent from the interviews and focus group that there were instances where eligible patients were not approached. Some SRUs were more on-board (as reflected by willingness to recruit, etc.) than others, and this has been identified in other stroke rehabilitation studies [43]. On-site face-to-face training was delivered in addition to the required Good Clinical Practice (GCP) training. However, the use of a dedicated person (e.g. Research and Development or Clinical Research Network Staff) to screen and consent potential participants would help to minimise selection bias and reduce burden on SRU therapy staff. Full details about burden have not been reported due to word constraints.

Eligibility was a contentious topic for some. Physiotherapists identified that including both mRS Grades 4 and 5 encompassed a wide range of (dis) abilities and was open to interpretation. For instance some suggested that no patient within the NHS stroke unit environment is bedridden (mRS Grade 5) due to early mobilisation practices, and others reported that some mRS Grade 4 participants were “too good” because they were able to walk, albeit with assistance and short distances. This aligns with existing literature that substantial inter-rater variability is a limitation of the mRS [44]. The use of other approaches, such as the Rankin Focused Assessment (RFA) [45], can reduce this inter-rater variability [46–48], which should be considered in a future trial.

As a feasibility trial, no hypothesis testing of patient-centred outcomes was planned or undertaken. However, the within-group improvements and between-group mean differences and corresponding confidence intervals provide some evidence that the functional standing frame programme shows some promise, e.g., a mean 2-to-3-point increase in BI scores observed between baseline and each of the follow-up time-points. This is higher than the suggested ≥ 1.85 point minimal clinically important difference on the BI score for people with stroke [49].

Both proposed primary outcome measures (BI and Edmans) showed similar magnitude of improvement in scores at each time-point. The BI provides a total summed score; therefore it is not possible to identify improvements in its individual domains. Conversely, the Edmans provides separate scores for its individual domains. There is a paucity of data on the Edmans, which makes meaningful comparisons with other rehabilitation trials difficult. In contrast, the BI is used extensively in stroke trials [50], enabling meaningful comparisons with other stroke rehabilitation trials. Therefore, the feasibility trial suggests the BI could be used in a definitive trial. The reported variability in the outcome measures (Additional file 3) would help to inform a power calculation for a future trial.

It was feasible and acceptable to participants to be assessed at five time points: baseline, post-intervention period, and 15, 29 and 55 weeks. The Stroke Recovery and Rehabilitation Roundtable taskforce [51] has developed a framework encapsulating definitions of critical time-points linked to the current understanding of biological recovery in the first weeks-to-months post-stroke. They recommend assessing from hyper-acute to chronic (> 6 months) but do not explicitly suggest a final time-point beyond 6 months. Assessment at three months is considered essential for all stroke trials that are testing sensorimotor interventions, and at least 6 months for trials conducting an economic evaluation [51]. Although it was feasible and acceptable to participants to be followed-up at 12 months post-randomisation, the proportion of deaths between 6 and 12 months suggests that the final assessment point for a definitive trial should be at 6 months.

In stroke rehabilitation trials of early standing [17, 52, 53], PROMs have not been consistently used, thereby limiting the opportunity to capture quality of life data. This feasibility trial aimed to determine if people with severe sub-acute stroke (including those with moderate to severe cognitive and communication impairments) could complete self-report measures. Based on qualitative interviews and completion of PROMs, it was more acceptable to participants and blinded assessors and feasible to collect the EQ-5D-5L data (86.7%) than the SAQoL-39 (75.6%). This may be due to the smaller number of questions (6 versus 39 respectively). However, only 22.2% were able to complete the health state rating for the EQ-5D-5L, which required participants to score their health out of 100. Ability to assign a rating can be affected post-stroke due to reduced capacity of abstract thinking [54] as well as ability to write, point to or speak their response. Despite this, it is anticipated that the EQ-5D-5L would be the only PROM used in a definitive trial, which mirrors the recommendations of the Stroke Recovery and Rehabilitation Roundtable [51]. The EQ-5D-5l also has the advantage of enabling a health economic analysis to be conducted.

Adherence to the intervention was low. For some, the duration of supported standing was only 2 to 3 min per session, and no standing occurred during eight sessions. Reasons for not standing during these eight sessions were not captured.

It is not known whether low adherence of standing time and sit-to-stand repetitions was due to participant ability or adherence of physiotherapists to the protocol due to physiotherapist- or organisation-factors. Possible reasons for this include the lengthy preparation time required to assist someone with severe stroke into standing, or the large proportion of rest time during the session. Some participants reported they were exhausted during their sessions and were reliant on their physiotherapist to encourage and motivate them to continue their sessions. Conversely, others reported an internal motivation to “push through the tiredness”, declining to end a session when their physiotherapist suggested they stop. This aligns with existing literature reporting that patients do not necessarily mind being pushed to work hard during rehabilitation, recognising this can be helpful when their motivation is lagging [55]. The ReAcT study [56] identified multiple interlinked factors influencing therapy provision, many of which were identified in SPIRES. For example, patient factors such as fatigue and therapists’ beliefs about patients’ ability to tolerate therapy influenced the length of the therapy sessions. There were differences in the session duration based on age and stroke severity, with those aged ≥ 80 years and/or with severe stroke receiving less physiotherapy.

Adherence, defined as the degree to which the behaviour of trial participants corresponds to the intervention assigned to them [57], is a key variable influencing the outcome of clinical trials [58]. This definition suggests the responsibility of adherence lies with the patient and does not consider the severity of physical, cognitive, communication, psychological impairments. Therapists’ reported beliefs indicated that the trial protocol and procedures and evidence-based clinical guidelines impacted on the degree of adherence to the trial interventions. So too did organisational priorities (such as a discharge driven culture) and staffing levels impact on this. Consequently, people with severe sub-acute stroke were possibly denied the opportunity to engage fully in the trial interventions and procedures.

A range of barriers were identified to implementing the trial processes, many of which could be resolved with additional staff training. Whilst there are no recommendations as to what comprises effective training associated with delivery of a RCT, commonality exists in the barriers to trial success, such as clinical and personal equipoise, gatekeeping, the impact of clinicians’ beliefs and attitudes and unconscious bias [59, 60]. Web-based training for treating therapists, with multi-modal learning formats, has been used successfully in other rehabilitation trials [61, 62]. An advantage of web-based training is that it could keep training costs to a minimum without compromising quality and effectiveness. Peer support can be effective in clinical practice [61, 63] and might also translate into the research arena, although this has yet to be investigated. Development of a core set of standards for training in stroke rehabilitation trials could help optimise successful delivery.

Falls were the most common AE during the trial. However, falls during inpatient stroke rehabilitation are common [64, 65] and people with stroke fall at almost twice the rate compared with healthy aged-matched adults [66]. It is estimated that approximately 50% of people with stroke discharged home fall in the first year with up to 40% falling repeatedly [66, 67]. In SPIRES, only six falls occurred during the 3-week intervention (15%); 14 reported at week 15 (42%); 6 at week 29 (19%) and eight at week 55 (30%) and all falls occurred outside of intervention/control group sessions. Thus, prevalence of falls within SPIRES was below those reported elsewhere. However, in a definitive trial falls risk will be mitigated as far as practicable through local SRU policies and more in-depth monitoring of the precipitating factors underlying falls.

## Limitations

The primary limitation of this feasibility trial was that the adherence criteria for the intervention did not account for any graded progression in standing time or sit-to-stand repetitions for each of the 3 weeks, thereby clouding interpretation of the data. Further, fidelity checking was planned for 10% of recruited participants; observing 10% of total number of planned sessions would have identified issues early on and may have been dealt with by on-site training during the trial period. Furthermore, fidelity checking did not include formal measures of agreement and reproducibility; it is recommended that this be included in the definitive trial.

Given sample sizes and breadth of topics covered, it is possible there were insufficient interviews conducted to capture all relevant information. However, diversity in sampling [68] (patients, relatives, physiotherapists across sites) was achieved.

All SRUs were in the South West of England; therefore, we do not know whether behaviour and attitudes of physiotherapists, patients and relatives are representative of all SRUs. However, similar issues were evident in the various sites where the study was undertaken and are in line with those of other national and international trials such as AVERT [69].

## Conclusions

This trial provides evidence that evaluating the functional standing frame programme for people with severe sub-acute stroke is not feasible in its current design. However, solutions have been identified to enable progression to a clinical and cost-effectiveness trial.

## Supplementary Information

**Additional file 1.** Work Instruction for the intervention.

**Additional file 2: Table 1 and Table 2.** Adverse and Serious Adverse Events.

**Additional file 3: Table 3.** Completeness of data for proposed secondary patient report outcome measures.

**Additional file 4: Table 4.** Mean differences between baseline and 3, 15, 29 and 55 weeks for the primary outcome data.

**Additional file 5: Table 5.** Illustrative quotes from interviews and focus group.

**Additional file 6: Table 6.** Criteria for progression to full trial.

**Additional file 7: Figure 1.** Proportion of participant versus proxy responses for both proposed primary outcome measures [Graph].

## Data Availability

The SPIRES trial protocol and statistical analysis plan are publicly available on the trial website [21]. The datasets used and/or analysed during the current study are available from the corresponding author (Chief Investigator) on reasonable request. Individual participant data that underlie the results will be made available (after de-identification) on a controlled access basis, subject to suitable data sharing agreements. Requesters will be asked to complete an application form detailing specific requirements, rationale, and proposed usage. Requests will be reviewed by the CI and study sponsor, who will consider the viability and suitability of the request and the credentials of the requester. Where access to requested data is granted, requesters will be asked to sign a data sharing agreement. Requested data will be made available, along with supporting documentation (e.g., data dictionary) on a secure server or through other secure data transfer method.

## Abbreviations

ADL: Activities of daily living
AVERT: A Very Early Rehabilitation Trial
BI: Barthel Index
CDRF: Clinical Doctoral Research Fellowship
CI: Chief investigator
CONSORT: The Consolidated Standards of Reporting Trials
COREQ: Consolidated Criteria for Reporting Qualitative Research
CT: Computer tomography
ICA: Integrated Clinical Academic
ISRCTN: International Standard Randomised Controlled Trial Number
mRS: Modified Rankin Scale
NHS: National Health Service
NIHR: National Institute for Health Research
OH: Orthostatic hypotension
PI: Principal Investigator
RCT: Randomised controlled trial
SPIRES: Standing Practice In Rehabilitation Early after Stroke
SRU: Stroke Rehabilitation Unit
TiDIER: Template for intervention description and replication
UK: United Kingdom
