# Supplementary material for: Functional standing frame programme early after severe sub-acute stroke (SPIRES): a randomised controlled feasibility trial
Source: Pilot Feasibility Stud. 2022 Mar 3;8:50. doi: 10.1186/s40814-022-01012-4 (PMC8892736; doi:10.1186/s40814-022-01012-4)

**Work Instruction for the intervention**

**1.0 Purpose of this document**

This Work Instruction provides detailed instructions to achieve safety and consistency when implementing the functional standing frame programme and recording the content of the usual physiotherapy group for the SPIRES trial across the four sites.

**2.0 Definitions**

**Orthostatic hypotension**: a sustained drop in systolic blood pressure of at least 20mmHg and/or diastolic blood pressure of at least 10mmHg within three minutes of moving from supine or sitting into standing.

**Brief questionnaire**: This is a double-sided A4 sheet which contains visual analogue scales with pictures to ensure people with aphasia can use. The questionnaire asks participants to rate their perceived level of enjoyment, fatigue, effort and any aches or pains.

**Physiotherapy Intervention Recording Tool**: A checklist which physiotherapists can tick boxes to indicate specific activities undertaken during physiotherapy sessions for both the functional standing frame programme and usual physiotherapy interventions. For the functional standing frame programme, boxes will be provided for you to record the number of minutes the participant stood for, number of repetitions of sit-to-stand and any AEs.

**3.0 Scope**

This Work Instruction applies to all clinicians and support staff involved in the SPIRES trial in all four sites:

- Lanyon Stroke Rehabilitation Unit, Camborne and Redruth Community Hospital
- Woodfield Stroke Rehabilitation Unit, Bodmin Community Hospital
- Staples Ward Stroke Unit, Northern Devon District Hospital
- Skylark Stroke Rehabilitation Unit, Mount Gould Hospital

**4.0 Responsibilities**

The PIs and treating physiotherapists at each site are responsible for adhering to the Good Clinical Practice guidelines and undertaking the trial specific training delivered by the Chief Investigator, Angie Logan.

The Principal Investigators and treating physiotherapists at each site are responsible for the safe and appropriate use of the standing frame and ensuring all its component parts are fit and safe for purpose.

The Principal Investigators and treating physiotherapists at each site are responsible for ensuring each participant is medically stable and continues to meet the inclusion criteria before they undertake the functional standing frame programme.

**5.0 Specific Procedure**

Flow chart 1 demonstrates the procedure for implementing the functional standing frame programme for at least the first three sessions where monitoring of cardiovascular responses is required. This requires blood pressure to be taken and recorded whilst in bed (supine for at least 3 minutes) and taken again within 1 minute of being transferred into a chair. If no signs of orthostatic hypotension, the participant can be taken to the gym to begin their functional standing frame programme. If the participant demonstrates a drop in systolic blood pressure of at least 20mmHg and/or diastolic blood pressure of at least 10mmHg within three minutes of moving from supine or sitting into standing, please refer to the Orthostatic Hypotension protocol (Flowchart 3).

Flow chart 2 demonstrates the procedure for participants who have three consecutive blood pressure readings within the participant’s normal range (≥ 90/60 to 120/80) where orthostatic hypotension was initially present but now resolved, or for those that do not have orthostatic hypotension.

*Duration of standing*

Thirty minutes’ maximum, however, this should be graded. The aim is to incrementally increase this by 30% during every subsequent session until the 30 minutes is achieved. For example, if a participant stood initially for 7 minutes, then the subsequent session aims for a 9-minutes stand). However, if this is not achievable, then a shortened increase in time based on the participants’ ability should be implemented.

*Repeated sit-to-stand*

Aim for eight to 12 repetitions to facilitate strengthening, however, this should be graded. The aim is to incrementally increase this by 30% during every subsequent session until the maximum 12 is achieved. For example, if a participant achieved three sit-to-stands, then the subsequent session aims to achieve four. However, if this is not achievable, then a lower number of repetitions based on the participants’ ability should be implemented.

*Upper limb exercise and/or table top activities*

These can be any activities that involve the hemi-plegic/paretic upper limb or both upper limbs. For example, muscle activation techniques; sensory input; facilitation of functional reach in tasks such as taking a drink, coming hair, washing face, table top games such as dominoes, Connect 4.

*Reductions in postural support*

Physiotherapists should use their clinical reasoning to progress the amount of postural support provided to participants by reducing the hip and trunk strap tension during standing and/or eliminating of the electronic power lifter for sit-to-stand.

*Progression of participants*

Should participants improve to the extent where support from the standing frame is not required, then unsupported standing/ walking can be progressed outside of the frame to optimise physical recovery for the remainder of the 3-week intervention if indicated. Repeated sit-to-stand (8–12 repetitions) should, however, be continued throughout the 30-minute session.

*Use of foot sensors under feet*

Customised foot sensors will be inserted under participants’ feet. The aim is for the foot sensors to be used as biofeedback to encourage equal weight distribution during quiet standing and sit-to-stand. Participants are not expected to maintain equal weight distribution for the entire session, especially when undertaking table top activities. Physiotherapists will use their clinical reasoning to undertake any additional activities to facilitate equal weight distribution, and document this on the Physiotherapy Content Recording Tool. Physiotherapists will record the weight distribution during quiet standing at the beginning and end of each session.

Please go to the SPIRES trial website: (<https://www.plymouth.ac.uk/research/spires>) which will include videos demonstrating standing, examples of how to progress the programme using case scenarios, downloadable schema of suggested task-specific exercises/ activities, advice on safety issues, what to do in the event of AEs and “frequently asked questions”. It will also include trial details (e.g. background, rationale) and the CI’s contact details. This will complement this SOP and the verbal training and support provided by the CI. A checklist of training undertaken by each of the treating physiotherapists will be recorded.

**Recording the content of your physiotherapy sessions**

Please use the Physiotherapy Recording Tool (in the Case Report Form) to record the content of every session for both the functional standing frame programme and usual physiotherapy.


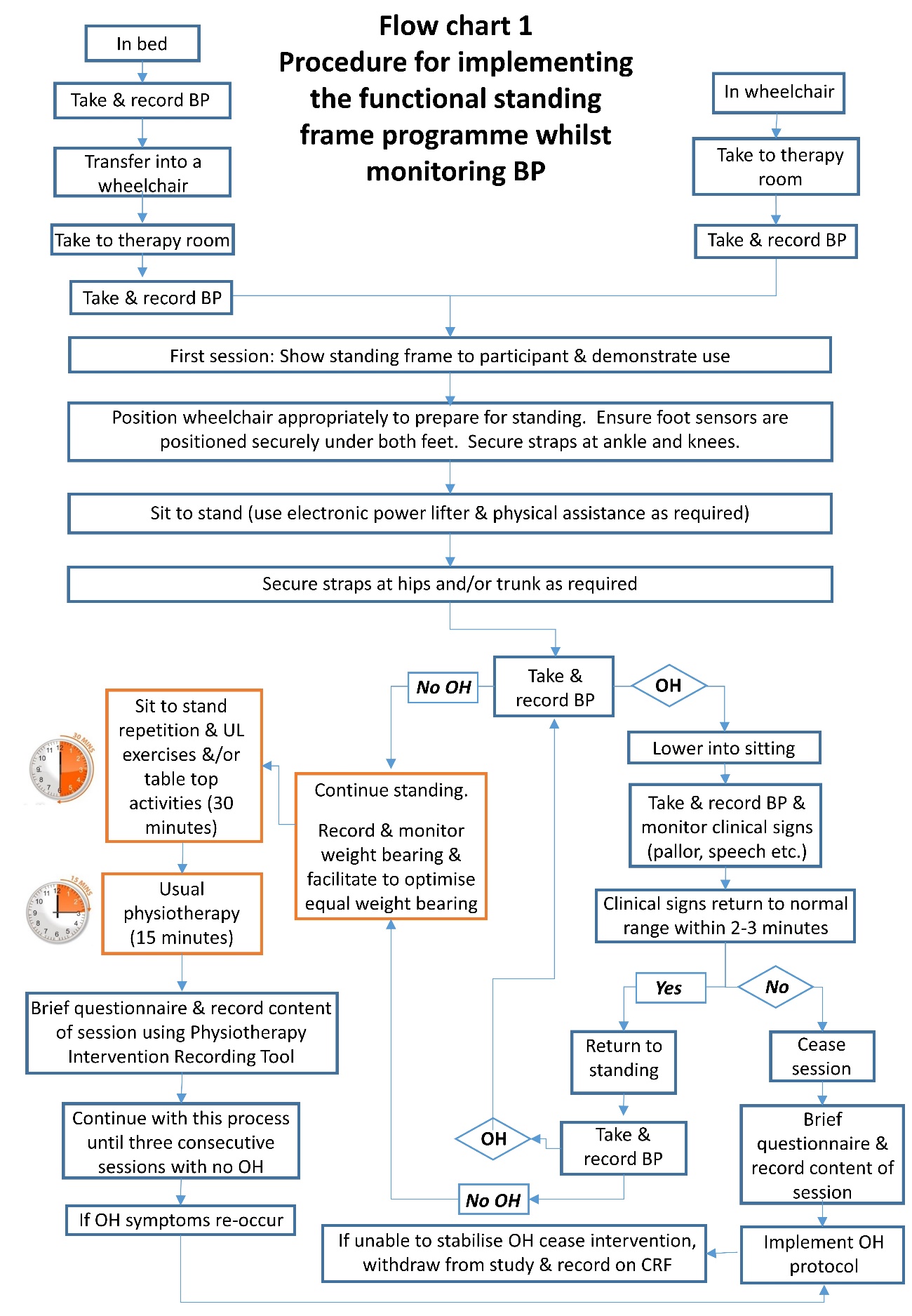


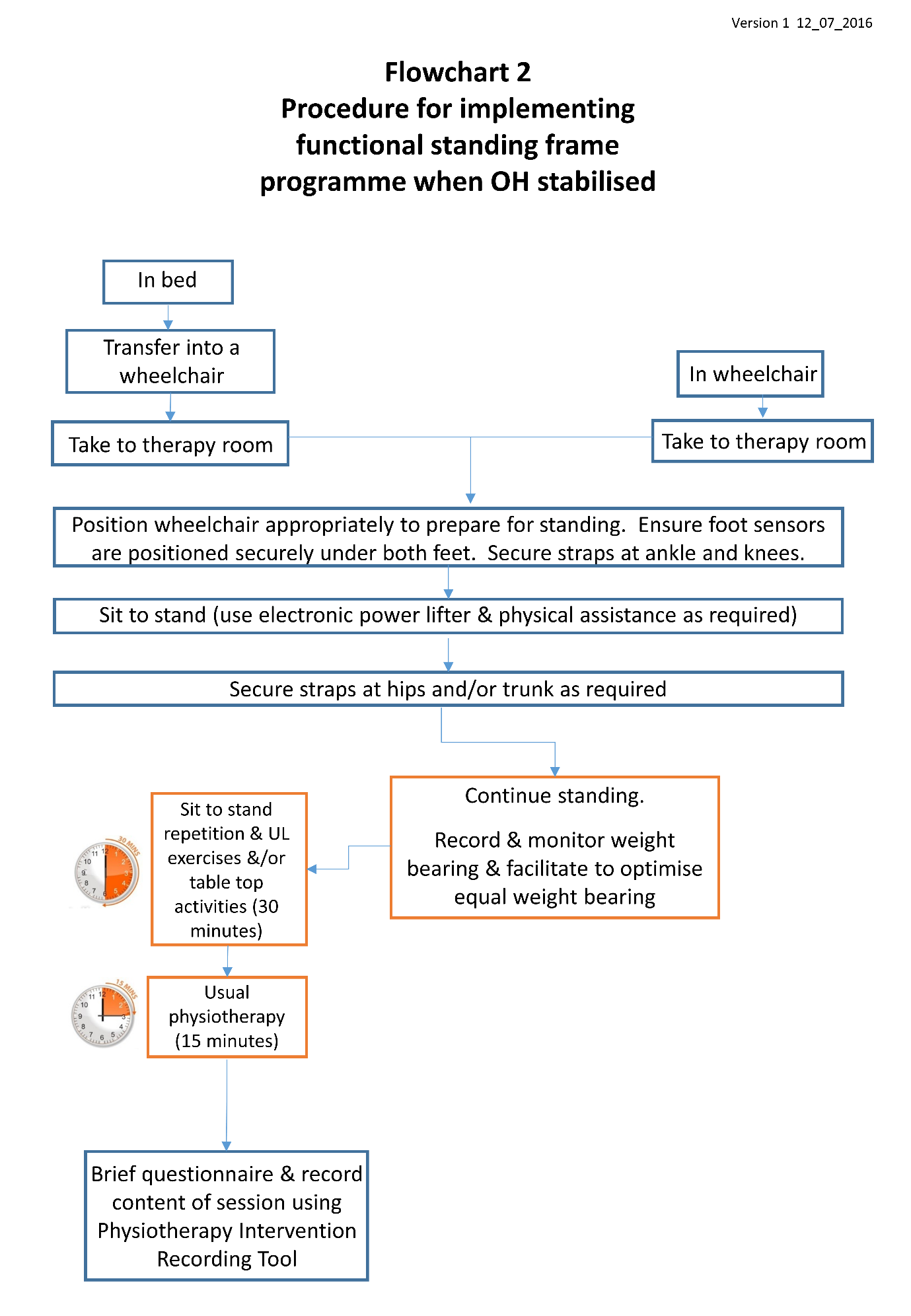


# **Flowchart 3 Orthostatic hypotension protocol**


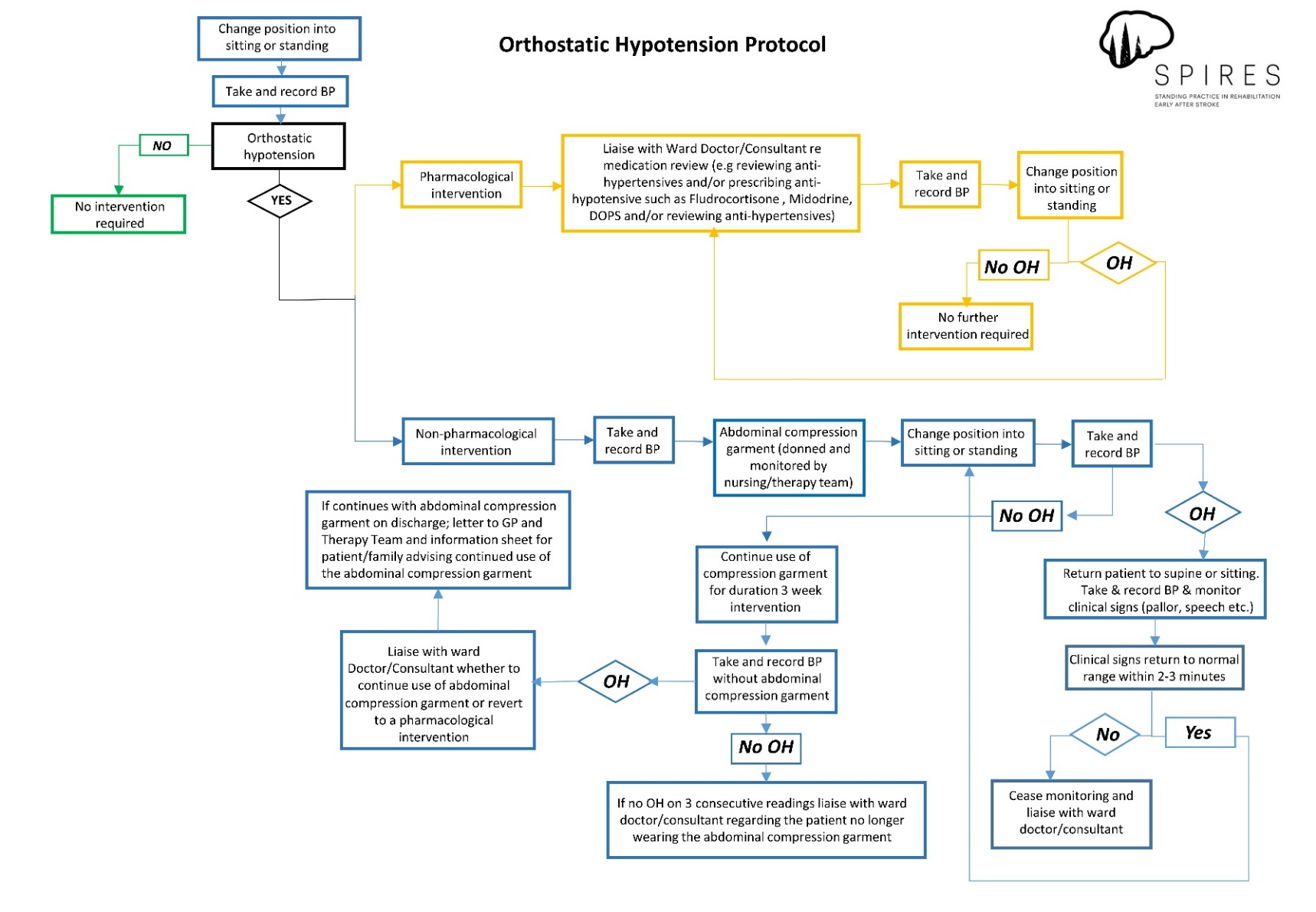

Supplement: Supplementary file 1 — Additional file 1. Work Instruction for the intervention. [file 40814_2022_1012_MOESM1_ESM.docx]
