# Supplementary material for: Functional standing frame programme early after severe sub-acute stroke (SPIRES): a randomised controlled feasibility trial
Source: Pilot Feasibility Stud. 2022 Mar 3;8:50. doi: 10.1186/s40814-022-01012-4 (PMC8892736; doi:10.1186/s40814-022-01012-4)
Supplement: Supplementary file 2 — Additional file 2: Table 1 and Table 2. Adverse and Serious Adverse Events. [file 40814_2022_1012_MOESM2_ESM.docx]

**Table 4** Adverse events in both groups

| **Organ system** | **Intervention count (n) and %** | | **Control**  **count (n) and %** | | |
| --- | --- | --- | --- | --- | --- |
|  | **Total AE*** **n=22** | **Total participants** n=45** | | **Total AEs*** **n=23** | **Total participants** n=45** |
| Blood and the lymphatic system disorders | 13.6 (3) | 4.4 (2) | | 4.3 (1) | 2.2 (1) |
| Cardiac disorders | 9.1 (2) | 4.4 (2) | | 0.0 (0) | 0.0 (0) |
| Congenital and familial and genetic disorders | 0.0 (0) | 0.0 (0) | | 4.3 (1) | 2.2 (1) |
| Ear and labyrinth disorders | 0.0 (0) | 0.0 (0) | | 4.3 (1) | 2.2 (1) |
| End of life care | 4.5 (1) | 2.2 (1) | | 0.0 (0) | 0.0 (0) |
| Fall | 68.2 (15) | 15.6 (7) | | 82.6 (19) | 20.0 (9) |
| Gastrointestinal disorders | 18.2 (4) | 6.7 (3) | | 8.7 (2) | 4.4 (2) |
| General disorders and admin site conditions | 0.00 (0) | 0.0 (0) | | 0.0 (0) | 0.0 (0) |
| Hepatobiliary disorders | 0.00 (0) | 0.0 (0) | | 0.0 (0) | 0.0 (0) |
| Infections and infestations | 22.7 (5) | 8.9 (4) | | 26.1 (6) | 11.1 (5) |
| Injury, poisoning and procedural complications | 4.5 (1) | 2.2 (1) | | 4.3 (1) | 2.2 (1) |
| Musculoskeletal and connective tissue disorders | 4.5 (1) | 2.2 (1) | | 4.3 (1) | 2.2 (1) |
| Nervous system disorders | 18.2 (4) | 8.9 (4) | | 13.0 (3) | 4.4 (2) |
| Orthostatic hypotension | 18.2 (4) | 6.7 (3) | | 0.0 (0) | 0.0 (0) |
| Psychiatric disorders | 4.5 (1) | 2.2 (1) | | 0.0 (0) | 0.0 (0) |
| Renal and urinary disorders | 27.3 (6) | 11.1 (5) | | 30.4 (7) | 8.9 (4) |
| Respiratory, thoracic and mediastinal disorders | 40.9 (9) | 13.3 (6) | | 60.9 (14) | 13.3 (6) |
| Skin and subcutaneous tissue disorders | 9.1 (2) | 4.4 (2) | | 13.0 (3) | 6.7 (3) |
| Surgical and medical procedures | 4.5 (1) | 22.2 (1) | | 0.0 (0) | 0.0 (0) |
| **Total (n)** | **59 AEs** | **20* participants** | | **59 AEs** | **18* participants** |

Most participants had more than one AE reported in the same or different organ system therefore this number does not equal the total number in this column

*Calculated by number of AEs divided by total number of participants in this group **Calculated by number of people with AEs divided by total number of participants

Table 5 Serious adverse events in both groups

| **Organ system** | **Intervention count (n) and %** | | | **Control count (n) and %** | | |
| --- | --- | --- | --- | --- | --- | --- |
|  | **Total SAE *** **n=22** | **Total participants** n=45]** | **Death count** | **Total SAEs*** **n=23** | **Total participants**** **n=45** | **Death count** |
| Endocrine system | 0.0 (0) | 0.0 (0) |  | 4.3 (1) | 2.2 (1) |  |
| Fall | 4.5 (1) | 2.2 (1) |  | 0.0 (0) | 0.0 (0) |  |
| Gastrointestinal disorders | 9.1 (2) | 4.4 (2) |  | 0.0 (0) | 0.0 (0) |  |
| General disorders and admin site conditions | 4.5 (1) | 2.2 (1) | 4.5 (1) | 4.3 (1) | 2.2 (1) | 4.3 (1) |
| Infections and infestations | 31.8 (7) | 11.1 (5) | 18.2 (4) | 21.7 (5) | 11.1 (5) | 4.3 (1) |
| Injury, poisoning and procedural complications | 9.1 (2) | 4.4 (2) |  | 8.7 (2) | 4.4 (2) |  |
| Nervous system disorders | 9.1 (2) | 4.4 (2) | 4.5 (1) | 8.7 (2) | 6.6 (3) | 8.7 (2) |
| Respiratory, thoracic and mediastinal disorders | 4.5 (1) | 2.2 (1) |  | 4.3 (1) | 2.2 (1) | 4.3 (1) |
| Unknown | 4.5 (1) | 2.2 (1) | 4.5 (1) | 0.0 (0) | 0.0 (0) |  |
| **Total (n)** | **17 AEs** | **12**† **participants** | **7** | **13 AEs** | **13**† **participants** | **5** |
| Number of deaths during 3-week treatment period | |  | 9.0 (2) |  |  | 0.0 (0) |
| Number of deaths during follow-up period | |  | 22.7 (5) |  |  | 21.6 (5) |

†Seven participants had more than one SAE reported in the same or different organ system therefore this number does not equal the total number in this column

*Calculated by number of AEs divided by total number of participants in this group **Calculated by number of people with AEs divided by total number of participants
