# Supplementary material for: Functional standing frame programme early after severe sub-acute stroke (SPIRES): a randomised controlled feasibility trial
Source: Pilot Feasibility Stud. 2022 Mar 3;8:50. doi: 10.1186/s40814-022-01012-4 (PMC8892736; doi:10.1186/s40814-022-01012-4)
Supplement: Supplementary file 3 — Additional file 3: Table 3. Completeness of data for proposed secondary patient report outcome measures. [file 40814_2022_1012_MOESM3_ESM.docx]

**Table 6** Completeness of data for proposed secondary patient report outcome measures

| **Outcome Variable** | **Time point** | **Completeness of Outcome Measure out of 45 participants % (n)** | | **% (n) missing** | |
| --- | --- | --- | --- | --- | --- |
|  |  | Intervention (n=22) | Usual physiotherapy (n=23) | Intervention | Usual physiotherapy |
| **PHQ-9** | Baseline | 77.3 (17) | 82.6 (19) | 22.7 (5) [completed SAD-Q10] | 17.4 (4) [completed SAD-Q10] |
|  | Week 3 | 72.7 (16) | 87.0 (20) | 27.3 (6) [n=1 completed SAD-Q10, n=1 unwell, n=4 withdrawn] | 13.0 (3) [n=1 completed SAD-Q10 [n=2 withdrawn] |
|  | Week 15 | 72.7 (16) | 73.9 (17) | 27.3 (6) [all withdrawn] | 26.1 (6) [n=1 unavailable, n=5 withdrawn] |
|  | Week 29 | 59.1 (13) | 69.6 (16) | 40.9 (9) [n=3 completed SAD-Q10, n=6 withdrawn] | 30.4 (7) [all withdrawn] |
|  | Week 55 | 50.0 (11) | 65.2 (15) | 50.0 (11) [n=1 completed SAD-Q10, [n=10 withdrawn] | 34.8 (8) [all withdrawn] |
| **SAD-Q10**  This measure was only completed if participants were unable to complete the PHQ-9 due to aphasia | Baseline | 22.7 (5) | 17.4 (4) | 72.3 (17) | 82.6 (19) |
|  | Week 3 | 4.5 (1) | 4.3 (1) | 72.7 (16) | 86.9 (20) |
|  | Week 15 | 0.0 (0) | 0.0 (0) | 100.0 (22) | 100.0 (23) |
|  | Week 29 | 13.6 (3) | 0.0 (0) | 86.4 (19) | 100.0 (23) |
|  | Week 55 | 4.5 (1) | 0.0 (0) | 95.5 (21) | 100.0 (23) |
| **SAQoL-39**  Physical score | Baseline | 72.7 (16) | 78.3 (18) | 27.3 (6) | 21.7 (5) |
|  | Week 3 | 63.6 (14) | 73.9 (17) | 36.4 (8) | 26.1 (6) |
|  | Week 15 | 68.2 (15) | 73.9 (17) | 31.8 (7) | 26.1 (6) |
|  | Week 29 | 63.6 (14) | 69.6 (16) | 36.4 (8) | 30.4 (7) |
|  | Week 55 | 50.0 (11) | 65.2 (15) | 50.0 (11) | 34.8 (8) |
| **SAQoL-39**  Communication score | Baseline | 72.7 (16) | 78.3 (18) | 27.3 (6) | 21.7 (5) |
|  | Week 3 | 63.6 (14) | 73.9 (17) | 36.4 (8) | 26.1 (6) |
|  | Week 15 | 68.2 (15) | 73.9 (17) | 31.8 (7) | 26.1 (6) |
|  | Week 29 | 63.6 (14) | 69.6 (16) | 36.4 (8) | 30.4 (7) |
|  | Week 55 | 50.0 (11) | 65.2 (15) | 50.0 (11) | 34.8 (8) |
| **SAQoL-39**  Psychosocial score | Baseline | 72.7 (16) | 78.3 (18) | 27.3 (6) | 21.7 (5) |
|  | Week 3 | 63.6 (14) | 73.9 (17) | 36.4 (8) | 26.1 (6) |
|  | Week 15 | 68.2 (15) | 73.9 (17) | 31.8 (7) | 26.1 (6) |
|  | Week 29 | 63.6 (14) | 69.6 (16) | 36.4 (8) | 30.4 (7) |
|  | Week 55 | 50.0 (11) | 65.2 (15) | 50.0 (11) | 34.8 (8) |
| **SAQoL-39**  Energy score | Baseline | 72.7 (16) | 78.3 (18) | 27.3 (6) | 21.7 (5) |
|  | Week 3 | 63.6 (14) | 73.9 (17) | 36.4 (8) | 26.1 (6) |
|  | Week 15 | 68.2 (15) | 73.9 (17) | 31.8 (7) | 26.1 (6) |
|  | Week 29 | 63.6 (14) | 69.6 (16) | 36.4 (8) | 30.4 (7) |
|  | Week 55 | 50.0 (11) | 65.2 (15) | 50.0 (11) | 34.8 (8) |
| **EQ-5D-5L**  Mobility | Baseline | 81.8 (18) | 91.3 (21) | 18.2 (4) | 8.7 (2) |
|  | Week 3 | 63.6 (14) | 82.6 (19) | 36.4 (8) | 17.4 (4) |
|  | Week 15 | 68.2 (15) | 73.9 (17) | 31.8 (7) | 26.1 (6) |
|  | Week 29 | 63.6 (14) | 69.6 (16) | 36.4 (8) | 30.4 (7) |
|  | Week 55 | 50.0 (11) | 65.2 (15) | 50.0 (11) | 34.8 (8) |
| **EQ-5D-5L**  Self-care | Baseline | 81.8 (18) | 91.3 (21) | 18.2 (4) | 8.7 (2) |
|  | Week 3 | 63.6 (14) | 82.6 (19) | 36.4 (8) | 17.4 (4) |
|  | Week 15 | 68.2 (15) | 73.9 (17) | 31.8 (7) | 26.1 (6) |
|  | Week 29 | 63.6 (14) | 69.6 (16) | 36.4 (8) | 30.4 (7) |
|  | Week 55 | 50.0 (11) | 65.2 (15) | 50.0 (11) | 34.8 (8) |
| **EQ-5D-5L**  Usual activities | Baseline | 81.8 (18) | 91.3 (21) | 18.2 (4) | 8.7 (2) |
|  | Week 3 | 63.6 (14) | 82.6 (19) | 36.4 (8) | 17.4 (4) |
|  | Week 15 | 68.2 (15) | 73.9 (17) | 31.8 (7) | 26.1 (6) |
|  | Week 29 | 63.6 (14) | 69.6 (16) | 36.4 (8) | 30.4 (7) |
|  | Week 55 | 50.0 (11) | 65.2 (15) | 50.0 (11) | 34.8 (8) |
| **EQ-5D-5L**  Pain/Discomfort | Baseline | 81.8 (18) | 91.3 (21) | 18.2 (4) | 8.7 (2) |
|  | Week 3 | 63.6 (14) | 82.6 (19) | 36.4 (8) | 17.4 (4) |
|  | Week 15 | 68.2 (15) | 73.9 (17) | 31.8 (7) | 26.1 (6) |
|  | Week 29 | 63.6 (14) | 69.6 (16) | 36.4 (8) | 30.4 (7) |
|  | Week 55 | 50.0 (11) | 65.2 (15) | 50.0 (11) | 34.8 (8) |
| **EQ-5D-5L**  Anxiety/Depression | Baseline | 81.8 (18) | 91.3 (21) | 18.2 (4) | 8.7 (2) |
|  | Week 3 | 63.6 (14) | 82.6 (19) | 36.4 (8) | 17.4 (4) |
|  | Week 15 | 68.2 (15) | 73.9 (17) | 31.8 (7) | 26.1 (6) |
|  | Week 29 | 63.6 (14) | 69.6 (16) | 36.4 (8) | 34.8 (8) |
|  | Week 55 | 50.0 (11) | 65.2 (15) | 50.0 (11) | 34.8 (8) |
| **EQ-5D-5L**  Health State | Baseline | 77.3 (17) | 78.3 (18) | 22.7 (5) | 21.7 (5) |
|  | Week 3 | 63.6 (14) | 65.2 (15) | 36.4 (8) | 26.1 (6) |
|  | Week 15 | 63.6 (14) | 65.2 (15) | 36.4 (8) | 34.8 (8) |
|  | Week 29 | 45.5 (10) | 47.8 (11) | 54.5 (12) | 52.2 (12) |
|  | Week 55 | 36.4 (8) | 60.9 (14) | 63.6 (14) | 39.1 (9) |
