# Supplementary material for: Functional standing frame programme early after severe sub-acute stroke (SPIRES): a randomised controlled feasibility trial
Source: Pilot Feasibility Stud. 2022 Mar 3;8:50. doi: 10.1186/s40814-022-01012-4 (PMC8892736; doi:10.1186/s40814-022-01012-4)
Supplement: Supplementary file 4 — Additional file 4: Table 4. Mean differences between baseline and 3, 15, 29 and 55 weeks for the primary outcome data. [file 40814_2022_1012_MOESM4_ESM.docx]

Table 7 Mean differences between baseline and 3, 15, 29 and 55 weeks for the primary outcome data

|  |  | | **Timepoint** | | | | | | | | | | |  |  |
| --- | --- | --- | --- | --- | --- | --- | --- | --- | --- | --- | --- | --- | --- | --- | --- |
| **Outcome variable** | **Treatment Group** | | **Baseline summary**  **mean, SD (n=45)** | | | **3 weeks**  **mean, SD (n)** | | **15 weeks**  **mean, SD (n)** | | **29 weeks**  **mean, SD (n)** | | **55 weeks**  **mean, SD (n)** | |  |  |
| **Barthel Index** | | *Intervention* | | 2.32 (2.056) | 5.53, 5.29 (17) | | 7.0, 6.06 (16) | | 7.88, 6.88 (16) | | 8.33, 7.76 (12) | |  | | |
|  | | *Control* | | 2.57 (2.57) | 5.05, 4.67 (22) | | 6.82, 5.99 (17) | | 7.69, 6.08 (16) | | 7.47, 6.44 (15) | |  | | |
|  | | Mean difference [95% CI] | | - | 0.48  [-2.75, 3.72] | | 0.17  [-4.10, 4.45] | | 0.18  [-4.50, 4.88] | | 0.86  [-4.76, 6.49] | |  | | |

| **Edmans ADL Index for Stroke**  Washing total | *Intervention* | 0.73 (0.50) | 2.18, 2.53 (17) | 2.63, 2.90 (16) | 2.63, 3.40 (16) | 3.25, 3.77 (12) |
| --- | --- | --- | --- | --- | --- | --- |
|  | *Control* | 0.57 (0.66) | 1.77, 2.18 (22) | 2.35, 2.26 (17) | 2.63, 2.39 (16) | 2.33, 2.29 (15) |
|  | Mean difference[95% CI] | - | 0.41 [-1.13, 1.93] | 0.27 [-1.57, 2.11] | 0.00 [-2.12, 2.12] | 0.92 [-1.50, 3.33] |
| Grooming total | *Intervention* | 2.18 (2.99) | 4.41, 2.81 (17) | 5.00, 3.16 (16) | 4.50, 3.78 (16) | 4.83, 3.81 (12) |
|  | *Control* | 2.57 (3.10) | 4.23, 3.68 (22) | 5.06, 3.78 (17) | 5.63, 3.14 (16) | 4.93, 3.69 (15) |
|  | Mean difference [95% CI] | - | 0.18 [-1.99, 2.36] | -0.59 [-2.54, 2.43] | -1.13 [-3.63, 1.38] | -0.10 [-3.09, 2.89] |
| Dressing total | *Intervention* | 0.32 (0.72) | 1.82, 2.74 (17) | 1.81, 3.02 (16) | 2.13, 3.50 (16) | 3.08, 4.03 (12) |
|  | *Control* | 0.39 (0.72) | 1.45, 2.32 (22) | 2.00, 2.83 (17) | 2.13, 2.96 (16) | 2.47, 3.34 (15) |
|  | Mean difference [95% CI] | - | 0.37 [-1.28, 2.01] | -0.19 [-2.26, 1.89] | 0.00 [-2.34, 2.34] | 0.62 [-2.30, 3.53] |
| Meal times total | *Intervention* | 2.91 (3.01) | 5.06, 3.09 (17) | 6.06, 2.91 (16) | 6.38, 3.05 (16) | 6.83, 2.41 (12) |
|  | *Control* | 3.35 (3.28) | 5.14, 3.98 (22) | 6.94, 2.66 (17) | 6.44, 2.99 (16) | 6.53, 3.18 (15) |
|  | Mean difference [95% CI] | - | -0.08 [-2.41, 2.26] | -0.88 [-2.86, 1.10] | -0.63 [-2.24, 2.12] | 0.30 [-1.99, 2.59] |
| Basic mobility total | *Intervention* | 0.73 (0.76) | 2.59, 2.76 (17) | 3.19, 2.97 (16) | 3.94, 3.77 (16) | 3.92, 3.82 (12) |
|  | *Control*  Mean difference [95% CI] | 0.74 (1.05)  - | 2.05, 2.48 (22)  0.54 [-1.16, 2.24] | 3.19, 3.59 (17)  0.01 [-2.33, 2.36] | 3.50, 3.78 (16)  0.44 [-2.29, 3.16] | 4.13, 4.22 (15)  0.89 [-3.45, 3.02] |
| Advanced mobility total | *Intervention* | 0.04 (0.21) | 1.18, 2.35 (17) | 1.56, 3.05 (16) | 2.13, 3.34 (16) | 2.25, 3.49 (12) |
|  | *Control* | 0.04 (0.21) | 0.32, 1.09 (22) | 1.24, 1.86 (17) | 1.69, 2.70 (16) | 1.80, 2.43 (15) |
|  | Mean difference [95% CI] | - | 0.86 [-0.29, 2.00] | 0.33 [-1.45, 2.11] | 0.44 [-1.76, 2.63] | 0.45 [-1.90, 2.80] |
| Bed mobility total | *Intervention* | 0.14 (0.64) | 2.06, 2.76 (17) | 2.25, 3.32 (16) | 3.25, 4.07 (16) | 3.50 4.17 (12) |
|  | *Control* | 0.22 (0.74) | 1.68, 2.46 (22) | 2.47, 3.30 (17) | 3.00, 3.86 (16) | 3.93, 4.37 (15) |
|  | Mean difference [95% CI] | - | 0.38 [-1.31, 2.06] | -0.22 [-2.57, 2.13] | 0.25 [-2.62, 3.12] | -0.43 [-3.84, 2.98] |
| Kitchen activities total | *Intervention* | 0.05 (0.21) | 0.65, 1.97 (17) | 1.38, 3.07 (16) | 1.69, 3.24 (16) | 1.17, 2.73 (12) |
|  | *Control* | 0.05 (0.21) | 0.73, 1.67 (22) | 1.59, 2.87 (17) | 1.50, 2.53 (16) | 1.27, 2.25 (15) |
|  | Mean difference [95% CI] | - | -0.80 [-1.26, 1.10] | -0.21 [-2.33, 1.90] | 0.19 [-1.91, 2.29] | -0.10 [-2.07, 1.87] |
| Housework activities total | *Intervention* | 0.00 (0) | 0.41, 1.70 (17) | 1.13, 3.07 (16) | 1.06, 2.91 (16) | 0.92, 2.61 (12) |
|  | *Control* | 0.00 (0) | 0.09, 0.43 (22) | 0.18, 0.73 (17) | 0.31, 0.87 (16) | 0.33, 1.05 (15) |
|  | Mean difference [95% CI] | - | 3.21 [-0.44, 1.08] | 0.95 [-0.62, 2.51] | 0.75 [-0.80, 2.30] | 0.58 [-0.93, 2.10] |
