# Supplementary material for: Functional standing frame programme early after severe sub-acute stroke (SPIRES): a randomised controlled feasibility trial
Source: Pilot Feasibility Stud. 2022 Mar 3;8:50. doi: 10.1186/s40814-022-01012-4 (PMC8892736; doi:10.1186/s40814-022-01012-4)
Supplement: Supplementary file 5 — Additional file 5: Table 5. Illustrative quotes from interviews and focus group. [file 40814_2022_1012_MOESM5_ESM.docx]

Table 8 Illustrative quotes from interviews and focus group

| **Theme** | **Quotation** |
| --- | --- |
| 1. Organisational/cultural factors that impacted on trial implementation | *“It has all been a bit haywire recently as everyone has been off”* [Physio 2]  *“Here you’re under constant pressure to get people out, so you need to adapt and change their treatment in order to get them home, get them mobile and get them on their feet and safe for discharge.”* [Physio 7 FG]  *“I’m finding the 48 hours a little bit tricky to fit in, doing our initial assessment […] and all the paperwork that goes with that […] It feels like 72 hours would be better.”* [Physio 3]  *“I don’t think we’re necessarily in the culture of research and that’s quite difficult […] That whole kind of evidence-based approach isn’t thought of that highly, it’s quite a sweeping statement, but it’s not at the forefront of people’s minds.”* [Physio 5] |
| 1. Impact of stroke on participation in the trial | *“I’ll be honest I didn’t know if I was going to be alive in three weeks’ time. I felt so bad that I thought I might die” [PC3]*  *“It was energy zapping, mentally and physically […] I’ve been able to cope even though I’ve been tired” [PI2]*  *“They were so exhausted trying to get them up into standing and their BP dropping, they were too tired to do anything else like working on sitting and functional bed mobility.” [Physio 1]*  *“… so much else going on not really stroke related stuff. The stress of him having the stroke, not having a job, having to find our rent and probably not really stroke related stuff.” [R2]* |
| 1. Experience of trial procedures | *“I think it’s [inclusion criteria] just too broad […] The vast majority of our patients are a 4 [mRS score] and on the surface they look like they’re eligible for the trial, but actually they’re […] functionally too good for the standing frame.” [Physio 5]*  *“I think three weeks was a good amount to start with” [Physio 6]*  *“I don’t know that three weeks is long enough, but also again because of our length of stay and stuff, a lot of people are obviously going out into the community in that time, so if it could be continued in the community.” [Physio 1]*  *“[…] if you’re constrained to just do standing, I didn’t feel that my therapy, my physio sessions could evolve with my patient’s needs and changing needs and changing discharge plans.” [Physio 7 FG]*  *“We did get quite creative with the standing frame, like PADLs in the standing frame […] that then helped to involve our OT colleagues as well […] You can make it patient specific which is nice because obviously if it was rigid you wouldn’t be able to tailor it to your specific patients and that may have been a problem.” [Physio 5]* |
| 1. Patients’, relatives’ and physiotherapists’ experience of the functional standing frame intervention | *“I think as much as we tried to be creative, people often got bored […] and they weren’t necessarily enjoying it because they felt so scared” [Physio 5 FG]*  *“They weren’t tolerating it, they were getting really fatigued” [Physio 8 FG]*  *“The physio said to me the other day, do you want to stop now if you’re too tired, if you want to stop we can stop, I went “no”, […] let’s carry on you know.” [PI2]*  *“I'm in a position that I need to be pushed” [PC3]* |

**FG = focus group; Physio = physiotherapist; R = relative; PADLs = personal activities of daily living; OT = Occupational Therapist ; PC – patient control group, PI = patient intervention group**
