# Supplementary material for: Functional standing frame programme early after severe sub-acute stroke (SPIRES): a randomised controlled feasibility trial
Source: Pilot Feasibility Stud. 2022 Mar 3;8:50. doi: 10.1186/s40814-022-01012-4 (PMC8892736; doi:10.1186/s40814-022-01012-4)
Supplement: Supplementary file 6 — Additional file 6: Table 6. Criteria for progression to full trial. [file 40814_2022_1012_MOESM6_ESM.docx]

Table 9 Criteria for progression to full trial

|  | **Criteria** | **Scenario 1** | **Scenario 2** | **Scenario 3** | **Outcome** |
| --- | --- | --- | --- | --- | --- |
| 1 | % of recruitment target achieved (50 participants) | ≥70% of the target figure | 51-69% of the target figure | ≤50% of the target figure | 90% recruitment target achieved.  This criterion meets scenario 1 |
| 2 | Target figure = 75% of the percentage of participants randomised to the intervention group who participated in at least five sessions per week of the intervention (e.g. 30 minutes of standing, or a 30% increase in standing time every session, and 8-12 sit-to-stand repetitions). This includes an estimated dropout rate of 25% due to mortality[84, 85] | ≥70% of the target figure | 51-69% of the target figure | ≤50% of the target figure | 0% participants randomised to the intervention group participated in at least five sessions per week and within those five sessions completed the required standing time and sit-to-stand repetitions.  This criterion meets scenario 3. |
| 3 | Target figure = 60% of the percentage of participants randomised who completed their 29- and 55-weeks post-randomisation follow-up assessment. This includes an estimated 40% drop out rate due to mortality[84, 85]. | ≥70% of the target figure | 51-69% of the target figure | ≤50% of the target figure | 60% (27 participants) completed their 29- and 55-week follow-up assessment which equates to 100% of the criteria target.  This criterion meets scenario 1. |
| **Proposed action** | | **Proceed to submitting plan to funder for full trial** | **Discuss with TSC and funder about progression** | **No progression to a full trial in the current design** | **The trial will not progress without addressing adherence** |
