# Supplementary material for: Functional standing frame programme early after severe sub-acute stroke (SPIRES): a randomised controlled feasibility trial
Source: Pilot Feasibility Stud. 2022 Mar 3;8:50. doi: 10.1186/s40814-022-01012-4 (PMC8892736; doi:10.1186/s40814-022-01012-4)
Supplement: Supplementary file 7 — Additional file 7: Figure 1. Proportion of participant versus proxy responses for both proposed primary outcome measures [Graph]. [file 40814_2022_1012_MOESM7_ESM.docx]

**Figure 2** Proportion of participant versus proxy responses for both proposed primary outcome measures
